# Changing the mind: hypnosis and diabetes

**DOI:** 10.1590/1518-8345.0000.2868

**Published:** 2017-12-04

**Authors:** Maria da Graça Pereira

**Affiliations:** Associate External Editor of Revista Latino-Americana de Enfermagem, Associate Professor with Aggregation of Department of Applied Psychology, Escola de Psicologia, Universidade do Minho, Braga, Portugal. Email: gracep@psi.uminho.pt

**Figure:**
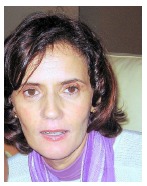

Diabetes, although a physiological disorder suffers the impact of negative psychological stresses through the dysfunctional activation of the autonomic nervous and endocrine systems. In fact, a variety of psychological variables have been found to be important in the metabolic control and management of diabetic patients, particularly regarding adherence to self-care behaviors and medication. Lifestyle therapy is the cornerstone of diabetes treatment and, therefore, any intervention that is able to achieve the control of glycaemia, prevent micro and macro complications of diabetes, improve patient’s quality of life and decrease diabetes risk factors is certainly welcome. 

Although acceptance of hypnosis and hypnotherapy by conventional medicine was officially acknowledged in 1958, by the American Medical Association and the Canadian Medical Association as a valid medical therapy, acceptance by health professionals has been slower. However, a substantial body of research has demonstrated the efficacy of hypnosis as part of the integrative treatment of many conditions that traditional medicine has found difficult to treat^1^ ^).^ In fact, hypnosis has shown not only to reduce anxiety in medical conditions but also change physiological parameters^2^ ^)^ and has been effective in the management of diabetes, including regulation of blood sugar, increased compliance, weight loss in adults as well as adolescents. Recently in an RCT, in type 1 diabetes patients, the hypnosis group decreased the standardized blood glucose levels, while the control group increased^3^ ^).^

One of the most problematic diabetic complications that is responsible for amputations is a diabetic foot ulcer and hypnosis has shown positive results in increasing peripheral blood circulation and decreasing diabetic foot problems. Also, mind-body interventions for vascular complications of diabetes have been reported to be effective in relieving diabetic angiopathy together with biofeedback. In general, the efficacy of hypnosis looks promising in diabetic foot care.

Hypnotic suggestions may indeed serve as a motivational coach and may be used effectively to alter beliefs and behaviors and hence serve as an ancillary method in the management of diabetes. The increased suggestibility shown during the hypnotic state would be helpful in increasing compliance for exercise, diet, and other lifestyle changes required for diabetes management, including stress reduction that is associated with the better metabolic control. Hypnosis may also be a helpful tool to induce relaxation, decrease psychological morbidity, often associated with diabetes, and accelerate wound healing, in case of diabetic foot ulcers. Another advantage of hypnosis is the fact that the patients could be taught self-hypnosis for home practice increasing patient’s sense of control over the disease, enhancing treatment outcomes. 

In conclusion, there is evidence that hypnosis can help people with diabetes because has shown to be a powerful method of helping people to change. This development might add diabetes to the list for which hypnosis may be an effective therapeutic adjunct. Recently, an approach called cognitive hypnotherapy^4^ that integrates clinical hypnosis within a model a cognitive psychotherapy has been proposed. This type of theoretical orientation is based on social constructivism in which language and meaning constitute the core ingredients of psychotherapeutic interaction with physically ill patients. Further research isneeded to assess the efficiency of this refinement of cognitive behavioral therapy.

In my viewpoint, the biggest challenge that hypnosis faces will probably be the dissipation of misconceptions and the implementation of scientific perceptions of hypnosis. The *Zeitgeist* may be ripe for an integrative medicine between the fields of allopathic medicine and hypnotherapy i.e. ready for a true appreciation of the interconnectedness of mind and body, in diabetic patient’s healing. We only hope that, hypnosis, together with other efficacious mind-body therapies may become an integral part of the health practice in a renewed collaboration between mental and physical health practitioners to optimally benefit patients.

## References

[1] Elkins G., Jensen M. P., Patterson D. R (2007). Hypnotherapy for the management of chronic pain. International Journal of Clinical and Experimental Hypnosis.

[2] Weisberg Mark B (2008). 50 years of hypnosis in medicine and clinical health psychology: A
synthesis of cultural Crosscurrents. American Journal of Clinical Hypnosis.

[3] Rodrigus F., Oliveira C., Silva C.F., D'almeida A (2017). Psychotherapy intervention with hypnosis in patients with type 1
diabetes mellitus. The European Proceedings of Social & Behavioural Sciences.

[4] Navon S (2014). The Illness/Non-Illness Model: Hypnotherapy for Physically Ill
Patients. America Journal of Clinical Hypnosis.

